# Molecular and serologic markers of HPV 16 infection are associated with local recurrence in patients with oral cavity squamous cell carcinoma

**DOI:** 10.18632/oncotarget.16747

**Published:** 2017-03-31

**Authors:** Chung-Guei Huang, Li-Ang Lee, Chun-Ta Liao, Tzu-Chen Yen, Shu-Li Yang, Yi-Chun Liu, Jung-Chin Li, Yu-Nong Gong, Chung-Jan Kang, Shiang-Fu Huang, Ku-Hao Fang, Kai-Ping Chang, Li-Yu Lee, Chuen Hsueh, Shin-Ru Shih, Kuo-Chien Tsao

**Affiliations:** ^1^ Department of Laboratory Medicine, Head and Neck Oncology Group, Linkou Chang Gung Memorial Hospital, Taoyuan, Taiwan, ROC; ^2^ Department of Medical Biotechnology and Laboratory Science, Chang Gung University, Taoyuan, Taiwan, ROC; ^3^ Graduate Institute of Biomedical Sciences, Chang Gung University, Taoyuan, Taiwan, ROC; ^4^ Faculty of Medicine, Chang Gung University, Taoyuan, Taiwan, ROC; ^5^ Department of Otorhinolaryngology - Head and Neck Surgery, Head and Neck Oncology Group, Linkou Chang Gung Memorial Hospital, Taoyuan, Taiwan, ROC; ^6^ Molecular Imaging Center, Head and Neck Oncology Group, Linkou Chang Gung Memorial Hospital, Taoyuan, Taiwan, ROC; ^7^ Department of Pathology, Head and Neck Oncology Group, Linkou Chang Gung Memorial Hospital, Taoyuan, Taiwan, ROC

**Keywords:** oral cavity squamous cell carcinoma, human papillomavirus, mRNA expression, serology, local recurrence

## Abstract

Human papillomavirus (HPV) infections predict mortality in Taiwanese patients with oral cavity squamous cell carcinoma (OCSCC). To address their prognostic significance for local recurrence (LR), in this retrospective cohort study we investigated different serologic and molecular markers of HPV 16 infection in 85 consecutive patients with primary OCSCC who received standard treatment and had their sera stored before treatment. Resected tumor specimens were examined with PCR-based assays for HPV 16 E6/E7 mRNA expression. Sera were tested with suspension arrays for the presence of HPV-specific antibodies using synthetic L1 and E6 peptides as well as a synthetic E7 protein. HPV 16 E6/E7 mRNA, anti-L1, anti-E6, and anti-E7 antibodies tested positive in 12%, 25%, 38%, and 41% of the study patients, respectively. Multivariate analysis identified pathological T3/T4, E6/E7 mRNA, and anti-E7 antibodies as independent risk factors for LR, whereas anti-E6 antibodies were an independent protective factor. In patients with ≥ 3 (high-risk group), 2 (intermediate-risk), and ≤ 1 (low-risk) independent risk factors (predictors), the 5-year LR rates were 75%, 42%, and 4%, respectively. Results were validated in an independent cohort. Together, our preliminary data indicate that HPV 16 infections as well as low and high serum levels of anti-E6 and anti-E7 antibodies, respectively, can serve as biomarkers of LR in patients with OCSCC, whereas the clinical usefulness of anti-HPV 16 antibodies for risk stratification of newly diagnosed cases deserves further scrutiny.

## INTRODUCTION

Oral cavity squamous cell carcinoma (OCSCC), a common malignancy of the head and neck, represents a significant public health concern in Taiwan and currently ranks third among all cancers in that country [1]. As of 2010, free screening for oral cavity cancer in Taiwanese individuals older than 30 years of age has been implemented. However, both the crude incidence and mortality rates of OCSCC are still increasing. In addition, the invasive nature of this malignancy continues to pose significant clinical challenges, especially with regard to local recurrence (LR).

Growing epidemiological evidence in Asia indicates that human papillomavirus (HPV) infections are involved in OCSCC tumorigenesis [2–3]. Based on PCR and *in situ* hybridization detection assays, HPV association is high for patients with OCSCC in Asia (odds ratio [OR]: 4.06; 95% confidence interval [CI]: 3.05–5.39) [2] and China (OR: 1.98; 95% CI: 1.34–2.92) [3] compared with those without cancer. HPV infections are common (overall rates of HPV-positive and HPV 16-positive tumors: 58% and 48%, respectively) in the Chinese population [4] although markedly less common (HPV and HPV 16: 19 and 8%, respectively) in Taiwan [5]. Based on serologic methods, 28% of patients with non-oropharyngeal cancer were HPV 16 seropositive (at least one among anti-L1, anti-E1, anti-E2, anti-E4, anti- E6, and anti-E7 antibodies) [6]. Recent studies have shown that HPV 16, which constitutes a high-risk HPV type, can play multiple roles in OCSCC including tumor progression [7], lymph node metastases [8], second primary malignancies [9], distant metastases [10–11], and mortality [5, 10]. In addition, although p16 expression is not significantly associated with HPV, p16 may mediate its effects by contributing to reduced proliferative capacity, leading to smaller tumor size and lower invasive potential in OCSCC [12]. However, to date, the majority of available studies focusing on HPV infections in patients with OCSCC have been on molecular profiling of tumor specimens. Conversely, only few data are currently available on the significance of immune response to HPV in patients with OCSCC. Specifically, serologic markers of HPV 16 infection (e.g., anti-L1, anti-E6, and anti-E7 antibodies) have been associated with the occurrence of oropharyngeal cancer [13] and higher anti-E6 titers at diagnosis are associated with increased risk of disease recurrence [14].

Because HPV 16 infections may induce immortalization of normal oral epithelial cells and promote *in vitro* progression of HPV-negative OCSCC by enriching cancer stemness [15], we hypothesized that they could be associated with LR after primary treatment. We therefore designed the current retrospective study with the main goal of determining the significance of both serologic and molecular markers of HPV 16 infection, as well as that of the gold standard status of mRNA for assignation of HPV oncogenic activity in head and neck cancer [16], in the prediction of 5-year LR rates in patients with OCSCC. We also determined whether different HPV 16 biomarkers could improve the prognostic stratification of patients with OCSCC subjected to radical surgery (either with or without adjuvant therapy).

## RESULTS

### General characteristics and treatment outcomes of patients

We included 85 patients (7 women and 78 men) in the study. Table 1 depicts the general characteristics of the study participants. The majority of study patients were older than 47 years and risky oral habits (alcohol drinking, betel quid chewing, and cigarette smoking) were common. In addition, most had well-to-moderately differentiated tumors, advanced stage disease (III/IV), a tumor depth ≥ 10 mm, and safe close margins (> 4 mm).

**Table 1:** General characteristics of the study patients and association between clinicopathological variables and 5-year local recurrence

Variablea	Number of Local recurrences	AUC of time-dependent ROC^b^	Local recurrence (*n* = 21)			
			Univariate analysis^c^	*P* value	Multivariate analysis^d^	*P* value
Sex		0.519				
Female (7 [8])	0		−		−	
Male (78 [92])	21		NA	NA	NA	NA
Age groups (years)		0.538				
≤ 47 (33 [39])	8		Reference		Reference	
> 47 (52 [61])	13		1.4 (0.6−3.2)	0.486	−	NA
Alcohol drinking		0.405				
Never (21 [25])	6		Reference		Reference	
Ever (64 [75])	15		0.8 (0.3−2.0)	0.588	−	NA
Betel quid chewing		0.422				
Never (23 [27])	5		Reference		Reference	
Ever (62 [73])	16		1.0 (0.4−2.8)	0.945	−	NA
Cigarette smoking		0.41				
Never (15 [18])	3		Reference		Reference	
Ever (70 [82])	18		1.2 (0.4−4.1)	0.757	−	NA
Differentiation		0.435				
Well/moderate (74 [87])	20		Reference		Reference	
Poor (11 [13])	1		0.3 (0.0−2.0)	0.196	−	NS
Pathological tumor status		0.571				
T1 + T2 (41 [48])	10		Reference		Reference	
T3 + T4 (44 [52])	11		1.4 (0.6−3.6)	0.338	2.9 (1.1−7.7)	0.035
Pathological nodal status		0.107				
N0 (39 [46])	12		Reference		Reference	
N1/ N2 (46 [54])	9		1.0 (0.4−2.3)	0.938	−	NA
Pathological stage		0.412				
I/II (27 [32])	7		Reference		Reference	
III/IV (58 [68])	14		1.1 (0.3−3.9)	0.835	−	NA
Pathological tumor depth (mm)		0.36				
< 10 (25 [29])	7		Reference		Reference	
≥ 10 [60 [71])	14		1.3 (0.5−3.2)	0.600	−	NA
Pathological close margin (mm)		0.356				
> 4 (54 [63])	13		Reference		Reference	
≤ 4 (31 [37])	8		1.0 (0.4−2.5)	0.937	−	NA
Bone invasion		0.474				
No (65 [76])	17		Reference		Reference	
Yes (20 [24])	4		1.0 (0.3−2.9)	0.934	−	NA
Skin invasion		0.5				
No (72 [85])	20		Reference		Reference	
Yes (13 [15])	1		0.5 (0.1−3.7)	0.488	−	NS
Perineural invasion		0.472				
No (48 [56])	13		Reference		Reference	
Yes (37 [44])	8		1.4 (0.6−3.4)	0.499	−	NS
Lymph invasion		0.471				
No (80 [94])	21		Reference		Reference	
Yes (5 [6])	0		0 (0−3152.1 )	0.588	−	NA
Vessel invasion		0.494				
No (84 [99])	21		Reference		Reference	
Yes (1 [1])	0		0 (0−3.0 × 10^4^)	0.656	−	NA
Extracapsular spread		0.459				
No (56 [66])	16		Reference		Reference	
Yes (29 [34])	5		1.0 (0.4−2.8)	0.994	−	NA
Level IV/V metastases		0.476				
No (81 [95])	21		Reference		Reference	
Yes (4 [5])	0		0 (0−1199.5)	0.555	−	NA
Treatment modality		0.451				
Operation (14 [17])	5		Reference		Reference	
Operation + RT (28 [33])	7		1.0 (0.3−3.1)	0.952	−	NA
Operation + CCRT (43 [51])	9		0.9 (0.4−2.5)	0.911	−	NA

AUC, area under the curve; CCRT, concomitant chemoradiotherapy; NA, not available; NS, not significant; ROC, receiver operating characteristic; RT, radiotherapy.

^a^Data are expressed as counts (%).

^b^Data are calculated based on ungrouped baseline data.

^c^Data are expressed as hazard ratios (95% confidence intervals) calculated with Cox regression models.

^d^Data are expressed as hazard ratios (95% confidence intervals) calculated with Cox regression models with a non-parametric 95% bootstrap confidence interval (200 runs). Significant *P* values are marked in bold.

After radical surgery, patients with advanced-stage cancer (*n* = 58), close margins (≤ 4 mm; *n* = 11), and/or ≥ 2 minor risk factors (*n* = 2) were treated with adjuvant radiotherapy or concomitant chemoradiotherapy as previously described [5, 17]. A total of 21 patients developed LR throughout the study period (median time to LR: 20 months, interquartile range [IQR]: 5−35 months). The 5-year LR rate of the entire discovery cohort was 36% (21/85; 95% CI: 23−49%). The 5-year disease-free survival (DFS), disease-specific survival (DSS), and overall survival (OS) rates of included patients were 43% (44/85; 95% CI: 31−54%), 61% (30/85; 95% CI: 49−72%), and 55% (34/85; 95% CI: 44−69%), respectively. In Taiwan, the 5-year OS rates in 2002−2006 and 2007−2011 were 47% and 52%, respectively [1], suggesting that our cohort was representative of the OCSCC population in this country.

### HPV 16 E6/E7 mRNA as an adverse prognostic factor for LR

**Figure 1:**
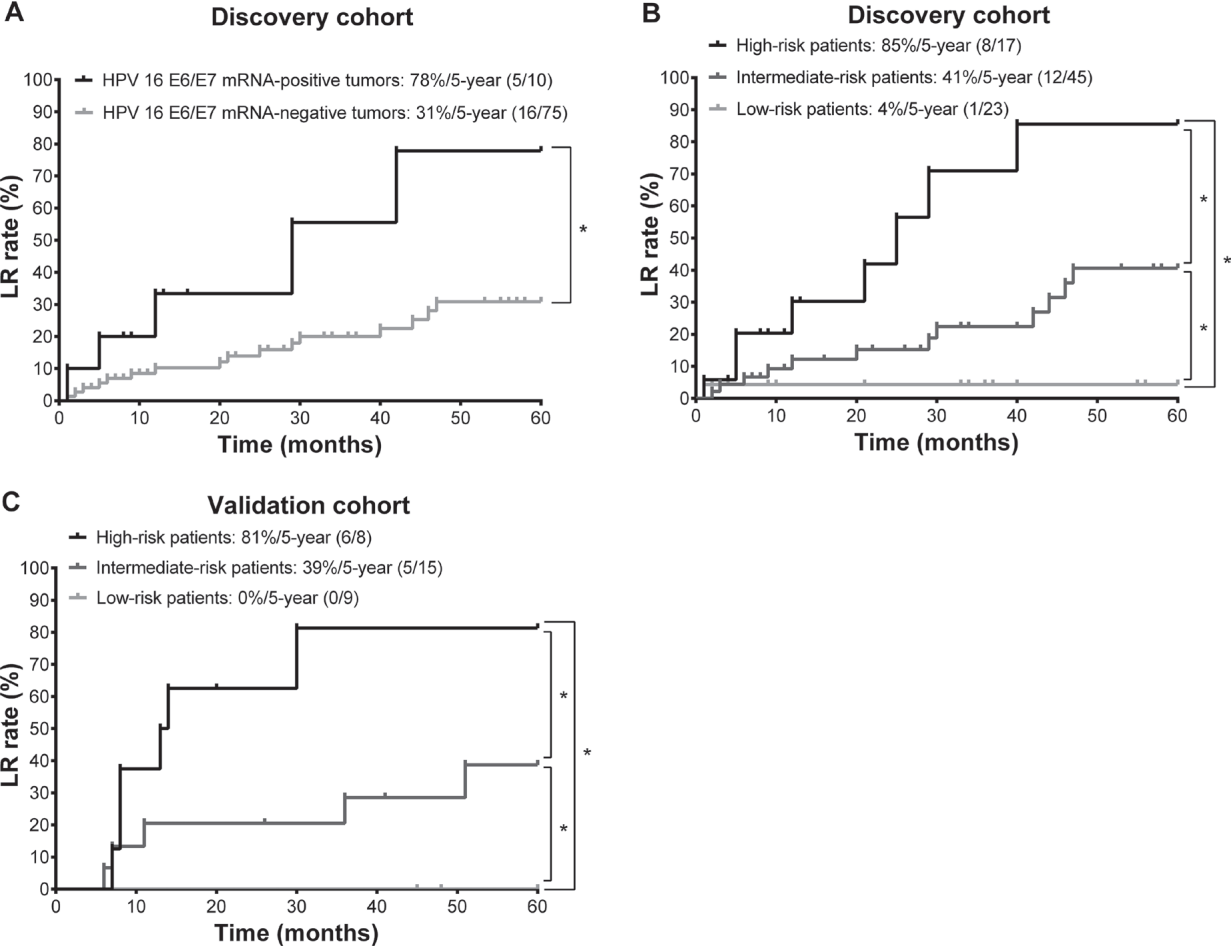
Local recurrence (LR) of 85 patients with oral cavity squamous cell carcinoma (OCSCC) (**A**) Kaplan-Meier curves depicting the 5-year LR rates of patients with OCSCC (the discovery cohort) stratified according to human papilloma virus (HPV) 16 E6/E7 mRNA status. (**B**) Kaplan-Meier estimates of 5-year LR rates in different risk groups (the discovery cohort). (**C**) Kaplan-Meier estimates of 5-year LR rates in different risk groups (the validation cohort). *P < 0.05 (log-rank test).

HPV 16 E6/E7 mRNA expression was detected in 12% (*n* = 10) of patients. Table 2 illustrates the associations of HPV 16 E6/E7 mRNA expression with certain clinicopathological factors related to LR such as betel quid chewing [18], tumor subsite; tumor differentiation; nodal status; tumor invasion ≥ 10 mm; margin distance ≤ 4 mm; bone invasion; skin invasion; perineural invasion; lymph invasion; vessel invasion [19–23]; and extracapsular spread [24]. We found that none of the differences in these variables of interest between HPV 16 E6/E7 mRNA-positive OCSCC and HPV 16 E6/E7 mRNA-negative OCSCC were statistically significant. With regard to tumor location, 50% (*n* = 5) of the HPV-positive tumors were localized in the cheek mucosa, 30% (*n* = 3) in the tongue, 10% (*n* =1) in the gum, and 10% (*n* = 1) in other anatomical sites (Table 2). The histological differentiation and tumor stage were largely similar in patients with HPV 16-positive and HPV 16-negative OCSCC. However, the 5-year LR rate of patients with HPV 16 E6/E7 mRNA-positive OCSCC was significantly higher (78%) than that of those with HPV 16 E6/E7 mRNA-negative OCSCC (21%; *P* = 0.008; log-rank test; Figure 1A).

**Table 2:** Frequency of human papillomavirus 16 E6/E7 mRNA-positive and human papillomavirus 16 E6/E7 mRNA-negative tumors

Variable	Human papillomavirus 16 E6/E7 mRNA-negative		Human papillomavirus 16 E6/E7 mRNA-positive		*P* value
	*N*	%	*N*	%	
Betel quid chewing					1.000
Never	20	27	3	30	
Ever	55	73	7	70	
Anatomical site					0.585
Tongue	28	37	3	30	
Cheek mucosa	29	39	5	50	
Gum	11	15	1	10	
Others	7	9	1	10	
Differentiation					0.348
Well/moderate	62	83	10	100	
Poor	13	17	0	0	
Pathological nodal status					0.748
N0	35	47	4	40	
N1/ N2	40	53	6	60	
Pathological tumor depth (mm)					1.000
< 10	22	29	3	30	
≥ 10	53	71	7	70	
Pathological close margin (mm)					1.000
> 4	27	36	4	40	
≤ 4	48	64	6	60	
Bone invasion					1.000
No	57	76	8	80	
Yes	18	24	2	20	
Skin invasion					1.000
No	63	84	9	90	
Yes	12	16	1	10	
Perineural invasion					0.741
No	43	57	5	50	
Yes	32	43	5	50	
Lymph invasion					0.474
No	71	95	9	90	
Yes	4	5	1	10	
Vessel invasion					1.000
No	74	99	10	100	
Yes	1	1	0	0	
Extracapsular spread					0.729
No	50	67	6	60	
Yes	25	33	4	40	

### Patients with high anti-E6 antibodies exhibit a low 5-year LR rate

**Figure 2:**
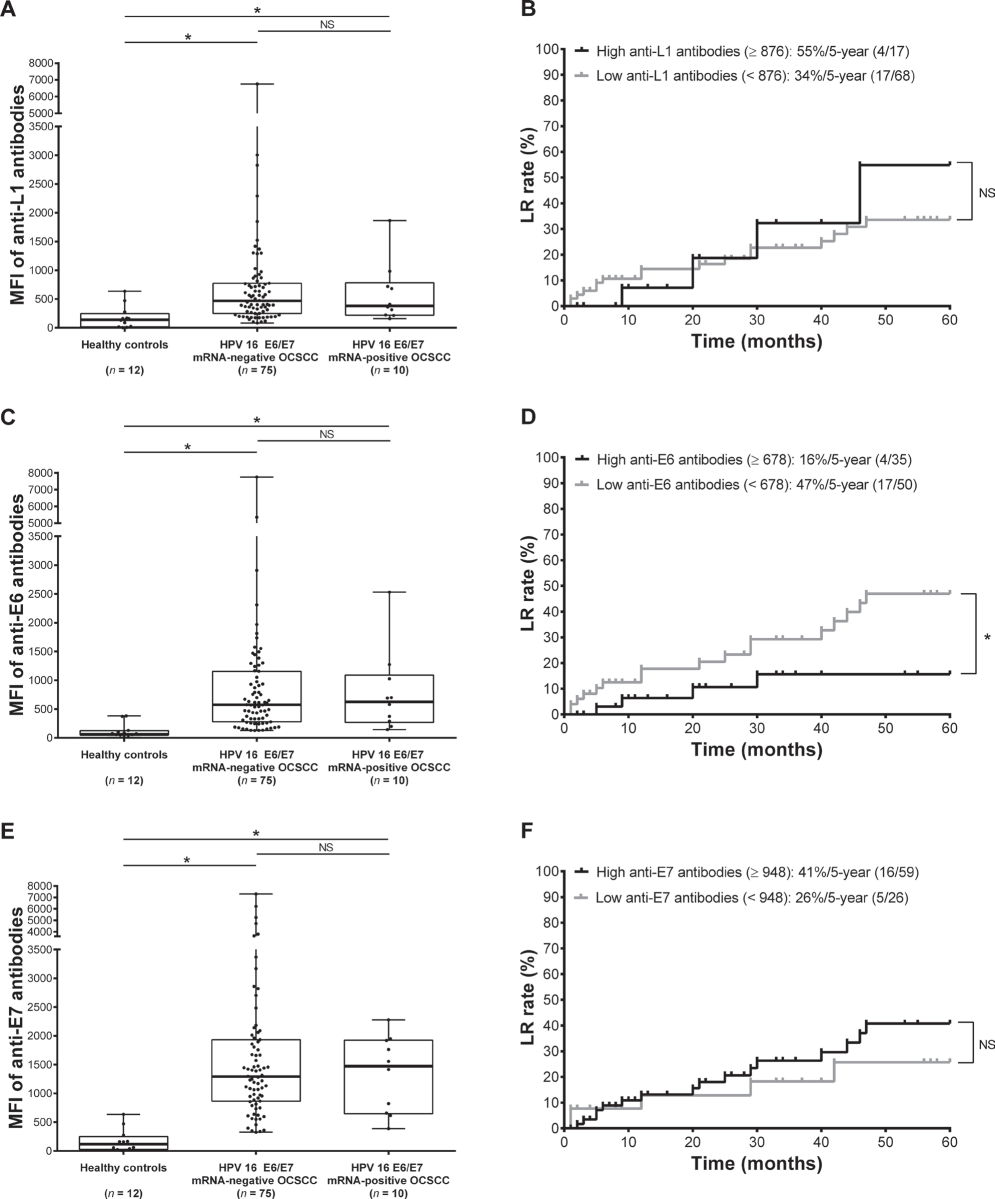
Serologic antibody profiles against human papilloma virus (HPV) 16 antigens and risk stratification in terms of 5-year LR rates (**A**, **C**, **E**) Plots representing the levels of anti-L1, E6, and E7 antibodies in 12 healthy controls, 75 HPV 16 E6/E7 mRNA-negative patients with oral cavity squamous cell carcinoma (OCSCC), and 10 HPV 16 E6/E7 mRNA-positive patients with OCSCC. The boundaries of the boxes indicate the interquartile ranges, whereas the lines in the boxes represent the medians. Whiskers indicate the maximal and minimal values. *P < 0.05 (Kruskal-Wallis test). (**B**, **D**, **F**) Kaplan-Meier curves depicting the 5-year LR rates of 85 patients with OCSCC stratified according to the optimal cutoffs of anti-L1, E6, and E7 antibodies. *P < 0.05 (log-rank test).

At enrollment, the median fluorescence intensity (MFI) values for anti-L1, anti-E6, and anti-E7 antibodies were 446 (interquartile range: 249−771), 580 (interquartile range: 284−1104), and 1315 (interquartile range: 838−1925), respectively. Patients with OCSCC (*n* = 85) had higher levels of anti-L1 (Figure 2A; *P* < 0.001; power = 0.87), anti-E6 (Figure 2C; *P* < 0.001; power = 0.93), and anti-E7 (Figure 2E; *P* < 0.001; power = 0.99) antibodies than healthy controls (*n* = 12). In contrast, levels of anti-L1, E6, and E7 antibodies did not differ significantly in patients with OCSCC and HPV E6/E7 mRNA-positive or HPV E6/E7 mRNA-negative tumors. The cutoff values for positivity were calculated from the means plus three standard deviations of data measured in all the healthy controls for the anti-L1 antibody and the means plus five standard deviations for anti-E6 and anti-E7 antibodies [25]. Specifically, the cutoff value of the anti-L1, anti-E6, and anti E7 antibodies were 769, 748, and 1447, respectively. The resulting prevalence rates of L1-, E6-, and E7-seropositivities were 25% (*n* = 21), 38% (*n* = 32), and 41% (*n* = 35).

Using time-dependent receiver operating characteristic (ROC) analysis, the areas under curve (AUC) were calculated for all tested serologic variables. The 5-year LR rates were similar in patients with OCSCC exhibiting high or low anti-L1 antibody levels (Figure 2B) as well as in those exhibiting high or low anti-E7 antibodies (Figure 2F). In contrast, the 5-year LR rate in patients with high anti-E6 antibodies was significantly lower than that in patients with low anti-E6 antibodies (Figure 2D). We then calculated the hazard ratios (HRs) with Cox regression analysis. Table 3 shows the associations between markers of HPV 16 infection and 5-year LR. Serum levels of anti-E6 antibodies showed an inverse association with 5-year LR, suggesting that their effect was protective. Neither anti-L1 nor anti-E7 antibodies were significantly associated with LR. Proportional hazard assumptions were met for all serologic variables (all *P* > 0.05).

**Table 3:** Association between molecular and serologic markers of human papillomavirus 16 infections and 5-year local recurrences

Variable^a^	Number of Local recurrences	AUC of time-dependent ROC^b^	Local recurrence (*n* = 21)			
			Univariate analysis^c^	*P* value	Multivariate analysis^d^	*P* value
E6/E7 mRNA		0.500				
Negative (75 [88])	16		Reference		Reference	
Positive (10 [12])	5		3.6 (1.3−10.0)	**0.014**	6.4 (2.0−20.0)	0.001
Anti-L1 antibodies		0.486				
≤ 876 (68 [80])	17		Reference		Reference	
> 876 (17 [20])	4		1.3 (0.4−3.8)	0.678	−	NA
Anti-E6 antibodies		0.369				
≤ 678 (50 [59])	17		Reference		Reference	
> 678 (35 [41])	4		0.3 (0.1−0.98)	**0.047**	0.2 (0.1−0.5)	**0.003**
Anti-E7 antibodies		0.512				
≤ 948 (26 [31])	5		Reference		Reference	
> 948 (59 [69])	16		1.6 (0.6−4.3)	0.389	3.9 (1.3−11.7)	**0.014**

AUC, area under the curve; NA, not available; NS, not significant; ROC, receiver operating characteristic.

^a^Data are expressed as counts (%).

^b^Data are calculated based on ungrouped baseline data.

^c^Data are expressed as hazard ratios (95% confidence intervals) calculated with Cox regression models.

^d^Data are expressed as hazard ratios (95% confidence intervals) calculated with Cox regression models with a non-parametric 95% bootstrap confidence interval (200 runs). Significant *P* values are marked in bold.

### Traditional clinicopathological variables are not significantly associated with 5-year LR

In univariate analysis, we failed to identify clinicopathological variables that were significantly associated with 5-year LR (Table 1). Proportional hazard assumptions were met for all clinicopathological variables (all *P* > 0.05).

### HPV 16 E6/E7 mRNA is not related to clinicopathological variables

Correlation analyses between pretreatment variables showed no associations of HPV 16 E6/E7 mRNA with clinicopathological variables of interest (Table 4). However, high anti-L1 antibodies were significantly associated with high anti-E6 antibodies. High anti-E6 antibodies were positively associated with high anti-L1 and E7 antibodies and inversely associated with age. With the exception of high anti-E6 antibodies, the presence of anti-E7 antibodies was not significantly associated with any other variables of interest.

**Table 4:** Association of markers of human papillomavirus 16 infection with the variables of interest

Variablea	Human papillomavirus 16 E6/E7 mRNA (positive)	High anti-L1 antibodies (> 876)	High anti-E6 antibodies (> 678)	High anti-E7 antibodies (> 948)
Human papillomavirus 16 E6/E7 mRNA (positive)	− (−)	< 0.001 (1.000)	0.065 (0.552)	−0.075 (0.498)
High anti-L1 antibodies (> 876)	< 0.001 (1.000)	− (−)	0.478 (< 0.001)	0.204 (0.061)
High anti-E6 antibodies (> 678)	0.065 (0.552)	0.478 (<0.001)	− (−)	0.400 (< 0.001)
High anti-E7 antibodies (> 948)	−0.075 (0.498)	0.204 (0.061)	0.400 (< 0.001)	− (−)
Age (> 47 years)	−0.098 (0.373)	0.035 (0.747)	−0.263 (0.015)	−0.079 (0.470)
Differentiation (poor)	−0.155 (0.156)	0.278 (0.010)	0.109 (0.319)	0.069 (0.529)
Pathological tumor status (T3 + T4)	−0.013 (0.907)	0.129 (0.238)	0.042 (0.701)	−0.130 (0.236)
Skin invasion (yes)	−0.054 (0.625)	0.196 (0.072)	0.043 (0.696)	−0.073 (0.509)
Perineural invasion (yes)	0.048 (0.665)	−0.024 (0.829)	−0.060 (0.588)	0.016 (0.882)

^a^Data are Spearman's rho correlation coefficients (*P* value). Significant *P* values are marked in bold.

### Prognostic factors for 5-year LR

Predictors with a *P* value < 0.50 (i.e., “E6/E7 mRNA”, “anti-E6 antibodies”, “anti-E7 antibodies”, “age”, “differentiation”, “pathological tumor status”, “skin invasion”, and “perineural invasion”) were entered into a regression model. “Age”, “differentiation”, “skin invasion”, and “perineural invasion” were manually removed when their regression coefficients did not show a meaningful sign in the first run. Although “anti-E6 antibodies” and “anti-E7 antibodies” were significantly associated with each other (Table 4), both were entered into the model during a second run to assess whether the results were consistent with our hypotheses. Finally, we used a bootstrapping approach (200 runs) for model shrinkage. The results indicated that a model that included “pathological tumor status”, “E6/E7 mRNA”, “anti-E6 antibodies”, and “anti-E7 antibodies” provided a significantly better fit than another model comprising “pathological tumor status”, “perineural invasion”, “E6/E7 mRNA”, “anti-E6 antibodies”, and “anti-E7 antibodies”.

The results of multivariate analyses identified four independent prognostic factors for 5-year LR: 1) HPV E6/E7 mRNA [HR = 6.4; 95% CI: 2.0−20.0; *P* = 0.001]; 2) high anti-E6 antibodies [HR = 0.2; 95% CI: 0.1−0.5; *P* = 0.003]; 3) high anti-E7 antibodies [HR = 3.9; 95% CI: 1.3−11.7]; *P* = 0.014; and 4) pathological T3 + T4 status [HR = 2.9; 95% CI: 1.1−7.7; *P* = 0.035; Table 2 and Table 3). With respect to risk stratification, anti-E6 antibodies ≤ 678 were reclassified as a risk factor in line with other prognosticators.

Based on these analyses, we stratified the 85 study patients (the discovery cohort) into three distinct risk groups based on the independent predictors identified in multivariate analysis, as follows: high-risk patients (≥ 3 predictors; *n* = 17), intermediate-risk patients (2 predictors; *n* = 45), and low-risk patients (0−1 predictor; *n* = 23; Figure 1B). As expected, the 5-year LR rates were significantly different in the three risk groups (85% vs. 41% vs. 4%, respectively, *P* < 0.001). The c-index was 0.71, suggesting a satisfactory predictive performance.

We then tested the accuracy of this model using a validation cohort of 32 additional patients with OCSCC. Age and sex of the validation cohort were comparable to those of the discovery cohort (age: 51 [IQR: 45–58] vs. 50 [IQR: 42–58], *P* = 0.485; women: 3% vs. 8%, *P* = 0.443). None of the proportions of HPV E6/E7 mRNA (12% *vs*. 13%; *P* = 1.000), high anti-E6 antibodies (41% *vs*. 38%; *P* = 0.833), high anti-E7 antibodies (69% *vs*. 81%; *P* = 0.249), pathological T3 + T4 status (52% *vs*. 44%; *P* = 0.535), or risk groups (20% [high risk]/53% [intermediate risk]/27% [low risk] *vs*. 25%/47%/28%; *P* = 0.799) were statistically significant. We found that this model performed fairly well in the validation cohort (Figure 1C; *P* = 0.002).

Although our stratification method did not significantly predict disease-specific survival (DSS; 44% *vs*. 61% *vs*. 72% in the three risk groups, respectively, *P* = 0.054), it was significantly associated with both 5-year DFS (16% *vs*. 41% *vs*. 61%, respectively, *P* = 0.006) and OS (38% *vs*. 53% *vs*. 72%, respectively, *P* = 0.023) rates.

## DISCUSSION

Consistent with our hypothesis, the current study demonstrates for the first time that HPV 16 infections, low serum levels of anti-E6 antibodies, and high serum concentrations of anti-E7 antibodies are independently associated with 5-year LR rates in patients with OCSCC. HPV 16 E6/E7 mRNA expression not only served as a molecular marker of HPV infection but also acted as an independent risk factor for LR. In addition, we demonstrated that high serum levels of anti-E6 antibodies may exert protective effects, being associated with a reduced likelihood of OCSCC regrowth after primary treatment. Conversely, increased concentrations of serum anti-E7 antibodies were a marker of risk for LR in OCSCC. Taken together, our preliminary findings suggest that the presence of an HPV 16 infection (as reflected both by molecular and serologic markers) may affect clinical outcomes in this malignancy.

LR rates of patients with head and neck cancer generally vary between 15 and 35%, with 90% of cases occurring within 3 years of primary treatment [26–27]. With the exception of buccal cancer arising in proximity of the cheek skin [28], a macroscopic margin of 1 cm appears to be adequate for surgical management of OCSCC [19]. Previous studies have shown that LR can be predicted by numerous clinicopathological variables including perineural spread; invasion to the lymphovascular system, bone, or muscle; tumor differentiation; tumor invasion ≥ 10 mm; margin distance < 5 mm; tumor size; nodal status; extracapsular spread; tumor subsite [19–23]; immunodepression [29]; and betel quid chewing [18]. With the exception only of tumor size, none of these variables were associated with LR in our study. This apparent discrepancy may be explained by the modest effect sizes of such risk factors in the pathogenesis of LR, which can yield negative findings in relatively small sample sizes. In contrast, the expression of HPV 16 E6/E7 mRNA in tumor specimens was significantly and independently associated with 5-year LR.

HPV 16 infections are involved in the early stages of oral carcinogenesis. In this regard, HPV 16 can only partially transform normal human oral keratinocytes into immortal cell lines *in vitro* [15] but is unable to cause tumor formation *in vivo* [30]. Notably, HPV 16 infections can be serologically identified over 10 years prior to tumor diagnosis [31]. Infections by high-risk HPV types have been associated with the development of invasive carcinomas in a subset of patients (17%) with oral epithelial dysplasia of the tongue [31]. In addition, both intralesional HPV infections [32, 33] and HPV 16 oral infections/seropositivity have been associated with oropharyngeal cancer [13, 25, 32–34] and OCSCC [7, 35] in case-control studies.

HPV status has been shown to predict survival in patients with oropharyngeal cancer [33, 36], with HPV-positive tumors having lower LR rates [27]. Compared with HPV-negative subjects, patients with HPV-positive oropharyngeal cancer have a significantly lower disease-specific mortality and are less likely to experience progression or recurrence [36]. HPV-positive patients with head and neck malignancies or oropharyngeal cancer and N2c disease demonstrate better clinical outcomes when treated with radiotherapy alone compared to more intensive chemoradiotherapy approaches. Consequently, an approach solely based on radiation therapy has been proposed for HPV-positive head and neck cancer [37, 38]. However, our current findings suggest that patients with HPV 16-associated OCSCC had worse 5-year LR rates following standard treatment compared to those without HPV 16 infections. Consistent with our data, recent studies show that patients with HPV-positive OCSCC had worse survival (*n*: 300–1002) than those with HPV-negative OCSCC in India [39], Iran [40], Croatia [41], and Taiwan [5, 9–11, 42]. Consequently, our data should caution against the reliance on radiotherapy alone as a general treatment strategy for patients with HPV-positive OCSCC. Moreover, the 5-year OS is only 16% when recurrences are discovered [43], with a rate of curative treatment as low as 5% in patients with advanced recurrences [27].

In particular, two potential mechanisms underlying the higher 5-year LR rates in patients with OCSCC who tested positive for HPV 16 E6/E7 mRNA expression merit comment. First, it is possible that HPV-positive OCSCC may be accompanied by field cancerization, although published evidence is conflicting. For example, HPV infection is not considered in some studies to be related to field cancerization or increased recurrence and the associated immune response as measured by lymphocyte infiltration may be considered protective in various related cancers such as oropharyngeal and tonsilar cancers [44–47], Conversely, other studies support the effects of field cancerization in HPV-positive tissue via factors such as smoking or betel nut chewing [9,18], potentially through additive genetic burden or additional changes in non-tumor-related tissues [48, 49].

A second possibility is that HPV 16 infections may promote OCSCC progression. The lack of significant associations between E6/E7 mRNA expression and other clinicopathological variables in our study cohort appears to suggest that HPV E6/E7 oncoprotein activation may play a direct role in LR. For example, the expression of E6 and E7 oncogenes may inactivate p53 and retinoblastoma (Rb), respectively. This would in turn result in a perturbation of cell cycle regulation in infected cells, ultimately promoting the onset of HPV-mediated carcinogenesis [50]. In addition, enhanced OCSCC stemness and poor OS may be associated with alteration in levels of specific miRNAs and their targets in conjunction with HPV 16/18 infection [15, 51]. Furthermore, HPV infection in e.g. head and neck cancers is associated with changes in the methylation profiles (or, increased methylation) of genes such as cyclin A1 that may in turn be related to LR and disease outcome [52–54]. We therefore hypothesize that HPV infections may promote LR by influencing multiple distinct molecular pathways. However, this possibility needs experimental validation in future studies, as HPV-infected human tissues express viral proteins at barely detectable levels and thus determining the ectopic expression of any given HPV gene remains challenging [55]. In addition, compared with HPV-driven head and neck cancer, epigenetic analysis of HPV-associated OCSCC has demonstrated fewer differences in DNA methylation profiles between HPV-positive and HPV-negative cases [56].

Despite the long-standing availability of HPV serology tests, most of the detection assays continue to remain for research use only. Previous studies demonstrated that the presence of HPV-specific antibodies against all antigens (L1, E6, E7, and virus-like particle) is strongly associated with HPV 16 DNA detectability in tumor tissues [34, 57–59] or oral lavage fluid [60]. Notably, anti-E6 and/or anti-E7 antibodies may be more specific than anti-virus-like particles as biomarkers of HPV-related head and neck cancer [57]. In oropharyngeal cancer, capsid antibodies (e.g., anti-L1 antibodies) are not expressed following tumor development and are lost when HPV DNA is integrated into host DNA (as their serum levels are relatively low) [61]. However, over 20% of Taiwanese patients with OCSCC exhibit high anti-L1 antibodies, which suggests that the difference in immune response may be either related to tumor compartment or ethnic diversity. Anderson *et al*. [34] highlighted the necessity of measuring multiple serologic markers of HPV16 infections; in this regard, some HPV16-positive tumors may be negative for E6 and E7 antibodies while testing positive for other antibodies. Furthermore, cellular immune responses specific to synthetic peptides from HPV 16 E6 and E7 oncoproteins are inversely related to recurrence-free survival in patients with HPV-positive cervical intraepithelial neoplasia [62], as well as to DSS and OS in head and neck cancer [60]. Here, we show that anti-E6 antibodies are inversely associated with LR, DSS, and OS in patients with OCSCC, thus possibly serving as a protective biomarker. In particular, the E6 protein binds to E6AP and then p53 to promote degradation of the latter to form mucosal patches containing genetically altered cells [63, 64], and/or binds to p300 to block p300-mediated p53 acetylation and activation [65]. Accordingly, high levels of anti-E6 antibodies may inhibit these mechanisms and reactivate p53 [65, 66], thereby reasonably reflecting a reduced likelihood of LR. These findings indicate that certain specific peptides may be useful as indicators of protective immunity.

Notably, anti-E7 antibodies can serve as either a protective factor [61] or a risk factor [67] for disease recurrence in patients with oropharyngeal cancer. The E7 protein inactivates the Rb pathway and causes cellar immortalization [68]. In addition, high anti-E7 antibodies may bind the E7 protein and reduce the likelihood of field expansion in mucosal patches [63]. Higher concentrations of anti-E7 antibodies at diagnosis have been associated with a significantly higher risk of recurrence in HPV-associated oropharyngeal cancer [67], a finding confirmed in our OCSCC study. Larger, longitudinal studies of HPV 16 serologic markers as prognostic variables in cancer patients are thus warranted to confirm and expand our results.

Our data support the clinical relevance of monitoring HPV infections in patients with OCSCC. In this regard, HPV vaccines can reduce the risk of malignant transformation in subjects at high risk for head and neck tumors [69]. Notably, mTOR inhibitors concurrent with standard-of-care chemoradiotherapy have been shown to increase cell killing and prolong survival in an animal model of HPV-positive head and neck cancer [70]. The question as to whether this approach could be clinically useful in patients with HPV-positive OCSCC therefore deserves further scrutiny.

Some caveats of our study merit comment. First, we did not investigate p16 expression, which is considered as the current standard for diagnosing HPV infections. However, our unpublished data suggest that p16 protein overexpression can rarely be identified (< 10%) in Taiwanese patients with HPV-positive OCSCC. Second, the inclusion of only patients treated with radical surgery may introduce selection bias influencing our results. Specifically, in our previous study a relatively improved tumor control and survival was observed in cases of buccal cancer with associated risk factors following radical surgery or broad surgical resection with neck dissection and post-operative radiotherapy or concomitant chemoradiotherapy [71]. However, fewer than 5% of patients with OCSCC underwent radiation therapy or neoadjuvant therapy as their primary treatment in many comprehensive cancer centers worldwide since 1990 [5, 72], thus limiting the availability of this alternative pool. Third, the retrospective nature of our study and the use of a convenience sample may limit the generalizability of our results. In the current study, the enrollment of a higher number of participants was not feasible and some important clinical factors such as sexual behavior, oral health, and nutrition status were not available. We nonetheless believe that our data are worth reporting because they can be considered hypothesis-generating and may stimulate further research in the field. Well-designed, prospective studies with more homogeneous sample sets and increased sample size are needed to confirm and expand our findings.

## MATERIALS AND METHODS

### Patients and samples

This retrospective cohort study was approved by the Institutional Review Board (IRB) of the Chang Gung Medical Foundation, Taoyuan, Taiwan (No. 99-3650B). All procedures were in compliance with the Helsinki Declaration of 1975. Written informed consent was obtained from all participants.

Between January 1, 2000 and December 31, 2009, blood samples (for serologic markers) and tumor specimens (for molecular markers) were collected from 278 Taiwanese patients with OCSCC who were consecutively treated at the Department of Otorhinolaryngology-Head and Neck Surgery (Chang Gung Memorial Hospital). The inclusion criteria were as follows: 1) newly diagnosed, histology-proven first primary OCSCC; 2) treatment with radical surgery with at least 1-cm gross safety margins (accompanied by neck dissection when required); 3) absence of suspected distant metastases on imaging; and 4) willingness to provide written informed consent. The following variables were collected from clinical charts: demographic data (sex, age at disease onset), risk factors for OCSCC (alcohol drinking, betel quid chewing, cigarette smoking), pathological tumor data (tumor subsite, differentiation, pathological T-status, pathological N-status, pathological stage, closest margin, tumor depth, extracapsular spread, level IV/V metastases), and patient status at the date of the last follow-up. Cancer staging was performed according to the 2002 American Joint Committee on Cancer sixth edition staging criteria [73]. Patients were excluded when the following criteria were met: 1) positive surgical margins (*n* = 12); 2) incomplete treatment (*n* = 2); 3) loss to follow-up within 5 years (*n* = 5); 4) insufficient or unusable paired pretreatment blood samples and tumor specimens (*n* = 151); 5) presence of HPV infections different from HPV 16 (*n* = 19); and 6) presence of multiple HPV infections (*n* = 4). Two PCR assays were used to screen HPV infections: 1) a commercially available HPV L1 gene PCR assay – EasyChip HPV Blot genotyping assay (King Car Ltd.) capable of detecting 39 distinct HPV types [5, 7, 9–11, 74]; and 2) an in-house real-time PCR assay for detecting HPV 16/18 E6 and E7 oncogenes [11].

**Figure 3:**
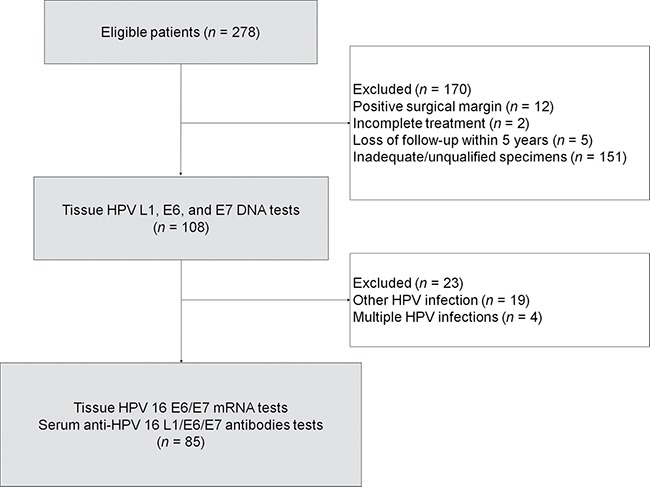
Flow of the patients through the study HPV, human papillomavirus.

Figure 3 depicts the flow of the participants through the study. Table 5 depicts the general characteristics of the study participants and shows the main differences between included and excluded cases. None of the differences reached statistical significance, the only exceptions being pathological tumor depth ≥ 10 mm (71% vs. 51%, respectively) and pathological close margins ≤ 4 mm (37% vs. 16%, respectively). For validation of our predictive model, we also collected a limited set of clinical and laboratory data on an additional 32 patients with OCSCC treated with the same protocol from January 1, 2010 to December 31, 2011 in another study [42].

**Table 5:** Baseline characteristics of included and excluded patients

Variable	Included (*n* = 85)	Excluded (*n* = 193)	*P* value
Sex (male/female)	78/7	187/6	0.07
Age (years)	50 (25−76)	50 (25−83)	0.24
Alcohol drinking (ever/never )	64/21	144/49	1.00
Betel quid chewing (ever/never)	62/23	157/36	0.15
Cigarette smoking (ever/never)	70/15	168/25	0.35
Differentiation (poor/well + moderate)	13/72	16/177	0.09
Pathological tumor status (T3 + T4 / T1 + T2)	44/41	101/92	1.00
Pathological nodal status (N1 + N2/ N0)	46/39	94/99	0.44
Pathological stage (III + IV/I + II)	58/27	134/59	0.89
Pathological tumor depth (≥ 10 mm/< 10 mm)	60/25	99/94	**0.004**
Pathological close margin (> 4 mm/≤ 4 mm)	54/31	163/30	**< 0.001**
Bone invasion (yes/no)	20/65	50/143	0.77
Skin invasion (yes/no)	13/72	19/174	0.22
Perineural invasion (yes/no)	37/48	80/113	0.79
Lymph invasion (yes/no)	5/80	13/180	1.00
Vessel invasion (yes/no)	1/84	3/190	1.00
Extracapsular spread (yes/no)	29/56	61/132	0.68
Level IV/V metastases (yes/no)	4/81	7/186	0.74

Data are expressed as counts or medians (ranges), as appropriate. Significant *P* values are marked in bold.

Formalin-fixed paraffin-embedded tumor specimens containing at least 10% tumor cells were prepared for DNA and mRNA extraction as previously described [5]. In brief, DNA was extracted using a Lab Turbo 48 automatic nucleic acid extraction system and a Lab Turbo Virus Mini Kit LVN500 (Taigen). Total RNA was isolated with the RNeasy FFPE Kit (Qiagen) [75]. Sample handling and PCR anti-contamination strategies have been reported previously [11].

Preoperative sera were collected from all patients with OCSCC at the time of admission (i.e., before treatment) [11, 76–78]. Serum was separated by centrifugation of blood samples (5 mL) at 1,940 × g (3000 rpm) for 10 min, aliquoted, and stored at −20°C in our Tissue Bank until immediately before analysis.

### Detection of HPV 16 E6/E7 mRNA

E6/E7 mRNA from HPV types 16, 18, 31, 33, and 45 was assayed using the NucliSENS EasyQ^®^ HPV kit (bioMérieux) under strict quality-check procedures [79]. The housekeeping *U1A* gene was used as an internal control. The assay was based on nucleic acid sequence-based-amplification real-time detection technology. The target area for primer design was the E6/E7 region. All of the primers and probes were designed to detect full-length transcripts. After amplification, the NucliSENS EasyQ system (bioMérieux) was used for routine analysis of experimental data.

### Measurement of anti-HPV 16 L1, E6, and E7 antibodies

The presence of IgG antibodies against antigens belonging to HPV-specific proteins was tested using an in-house suspension array technology. Synthetic peptides of HPV 16 L1 (N′-C-KHTPPAPKEDPLKK-C′; position: 456−471)/E6 (N′-C-RTAMFQDPQERPRK-C′; position: 5−18) and a recombination protein of full-length HPV 16 E7-histag fusion protein (N′-MHGDTPTLHEYMLDLQPETTDLYCYEQLNDSSEEEDEIDGPAGQAEPDRAHYNIVTFCCKCDSTLRLCVQSTHVDIRTLEDLLMGTLGIVCPICSQKP-C′; position: 1−98) were manufactured by CPC Scientific Inc. Peptide and protein antigenicity was confirmed by dot blotting and western blot. Toward this aim, anti-HPV 16 (05–134, National Institute for Biological Standards and Control) [80] and anti-HPV 18 (10–140, National Institute for Biological Standards and Control) sera were compared with sera obtained from healthy controls (without a known history of HPV infection and no previous vaccinations). The HPV 16 L1 peptide and E7 protein cross-reacted with the anti-HPV 16 and anti-HPV 18 sera, whereas the HPV 16 E6 peptide specifically reacted with the anti-HPV 16 serum. None of the sera obtained from 12 healthy controls reacted with HPV 16 L1, E6, or E7 antigens. In contrast, the reaction rates with these antigens for the 11 sera obtained from patients with HPV 16 L1 DNA-positive OCSCC were 81%, 54%, and 90%, respectively. In addition, two sera (100%) obtained from two patients with HPV 18 L1 DNA-positive OCSCC reacted with the HPV 16 E6 antigen but not with the HPV 16 L1 and E7 antigens. Carboxylated beads (1 × 10^6^; Bio-Rad) were conjugated with 6 μg bovine serum albumin (BSA)-conjugated peptides (synthetic HPV 16 L1/E6 peptides; CPC Scientific Inc.) or recombinant protein (full-length HPV 16 E7-His-tag fusion protein) as capture antigens of an amine coupling kit (Bio-Rad). Aliquots of coupled beads were combined in the assay buffer (1% BSA/PBS, pH 7.4) at a final concentration of 100 beads/μL analyte. A 50 μL aliquot of the multiplex beads solution (approximately 5,000 beads/analyte per well) was then added to each well of a 96-well filter plate (Millipore Corp.). Beads were incubated in solution (50 μL) with the following order: 1) sample diluent (1:50 dilution); 2) detection antibody (1:5000 dilution; biotin-labeled anti-human IgG antibody, Jackson ImmunoResearch Laboratories Inc.); and 3) streptavidin-PE (1:500 dilution, Jackson ImmunoResearch Laboratories Inc.). Wells were washed twice with 100 μL wash buffer (0.5% tween 20 /PBS, pH 7.4) after each incubation step. After final incubation and washing, beads were resuspended in 100 μL assay buffer before being assayed. A Bio-Plex 200 analyzer (Bio-Rad) was used to identify the internal color of individual beads and quantify their reporter fluorescence. The results of suspension arrays for each serum sample were expressed as the MFI of 100 beads per set. We further compared the serologic status of patients with HPV 16-positive OCSCC (based on the results of L1 DNA assays) with that of 12 healthy controls including 3 women and 9 men with a median age of 47 years (IQR, 40–54 years). Their sex and age were comparable to the discovery group (*P* = 0.106 and 0.490, respectively). Differences between the three anti-HPV antibodies were found to be significant (Figure 2).

### Statistical analysis

The main study endpoint was LR (defined as the time elapsed between primary surgery and histologically confirmed local tumor recurrence). Follow-up visits were continued until April 1, 2015. All of the patients received follow-up examinations for at least 60 months after surgery or until death. Subjects without a documented event were censored at the date of last follow-up. The patient baseline characteristics were compared using the Mann-Whitney *U* test, the chi-square test, or the Fisher's exact test, as appropriate. Cumulative LR rates were determined using the Kaplan-Meier method (log-rank test). The cutoffs characterized by the lowest *P* value on log-rank tests between the 10th and 90th percentiles were considered as optimal for levels of anti-HPV 16 L1, E6, and E7 antibodies (as well as for other variables). Cox time-dependent analysis was used to assess whether the proportional hazard assumption for each variable was met. We used Cox proportional hazard regression analysis with a bootstrap approach (200 runs) to identify variables associated with LR [81]. Results were expressed as HRs with their 95% CIs. Time-dependent ROC curves were used to assess the classification utility of continuous and ordinary variables [82]. The diagnostic accuracy for cumulative incidence was expressed by the AUC over the total observation period. We used a “forced simultaneous entry” approach and a “sign-correct” method for multivariate Cox proportional hazard regression models [83]. Variables that showed a *P* value < 0.50 in univariate analysis were entered as potential covariates in the multivariate model; however, they were removed when their regression coefficients did not show a meaningful sign. Variables that were not strongly related to other predictors (confounders) were removed to improve model precision [84]. Bootstrap validation was applied for model shrinkage [81, 83]. Patients were then categorized into distinct risk groups based on the number of independent risk factors. The discrimination ability of the model was assessed using the Harrell's c-statistic. All calculations were performed using the SPSS 23.0 statistical package for Windows (SPSS Inc.), R 3.2.2 software (R Foundation for Statistical Computing), and G*Power 3.1.9.2 software [85]. Two-tailed *P* values < 0.05 were considered statistically significant.

## References

[1] Chiang CJ, Lo WC, Yang YW, You SL, Chen CJ, Lai MS (2016). Incidence and survival of adult cancer patients in Taiwan, 2002–2012. J Formos Med Assoc.

[2] Shaikh MH, McMillan NA, Johnson NW (2015). HPV-associated head and neck cancers in the Asia Pacific: A critical literature review & meta-analysis. Cancer Epidemiol.

[3] Zhou J, Tao D, Tang D, Gao Z (2015). Correlation of human papilloma virus with oral squamous cell carcinoma in Chinese population. Int J Clin Exp Med.

[4] Zhu C, Ling Y, Dong C, Zhou X, Wang F (2012). The relationship between oral squamous cell carcinoma and human papillomavirus: a meta-analysis of a Chinese population (1994–2011). PLoS One.

[5] Lee LA, Huang CG, Tsao KC, Liao CT, Kang CJ, Chang KP, Huang SF, Chen IH, Fang TJ, Li HY, Yang SL, Lee LY, Hsueh C (2015). Human papillomavirus infections are common and predict mortality in a retrospective cohort study of Taiwanese patients with oral cavity cancer. Medicine (Baltimore).

[6] KA Lang Kuhs, Pawlita M, Gibson SP, Schmitt NC, Trivedi S, Argiris A, Kreimer AR, Ferris RL, Waterboer T (2016). Characterization of human papillomavirus antibodies in individuals with head and neck cancer. Cancer Epidemiol.

[7] Luo CW, Roan CH, Liu CJ (2007). Human papillomaviruses in oral squamous cell carcinoma and pre-cancerous lesions detected by PCR-based gene-chip array. Int J Oral Maxillofac Surg.

[8] Jitani AK, Raphael V, Mishra J, Shunyu NB, Khonglah Y, Medhi J (2015). Analysis of human papilloma virus 16/18 DNA and its correlation with p16 expression in oral cavity squamous cell carcinoma in North-Eastern India: A chromogenic in-situ hybridization based study. J Clin Diagn Res.

[9] Huang SF, Li HF, Liao CT, Wang HM, Chen IH, Chang JT, Chen YJ, Cheng AJ (2012). Association of HPV infections with second primary tumors in early-staged oral cavity cancer. Oral Dis.

[10] Lee LA, Huang CG, Liao CT, Lee LY, Hsueh C, Chen TC, Lin CY, Fan KH, Wang HM, Huang SF, Chen IH, Kang CJ, Ng SH (2012). Human papillomavirus-16 infection in advanced oral cavity cancer patients is related to an increased risk of distant metastases and poor survival. PLoS One.

[11] Huang CG, Lee LA, Tsao KC, Liao CT, Yang LY, Kang CJ, Chang KP, Huang SF, Chen IH, Yang SL, Lee LY, Hsush C, Chen TC (2014). Human papillomavirus 16/18 E7 viral loads predict distant metastasis in oral cavity squamous cell carcinoma. J Clin Virol.

[12] Satgunaseelan L, Virk SA, Lum T, Gao K, Clark JR, Gupta R (2016). p16 expression independent of human papillomavirus is associated with lower stage and longer disease-free survival in oral cavity squamous cell carcinoma. Pathology.

[13] D'Souza G, Kreimer AR, Viscidi R, Pawlita M, Fakhry C, Koch WM, Westra WH, Gillison ML (2007). Case-control study of human papillomavirus and oropharyngeal cancer. N Engl J Med.

[14] Fakhry C, Qualliotine JR, Zhang Z, Agrawal N, Gaykalova DA, Bishop JA, Subramaniam RM, Koch WM, Chung CH, Eisele DW, Califano J, Viscidi RP (2016). Serum antibodies to HPV16 early proteins warrant investigation as potential biomarkers for risk stratification and recurrence of HPV-associated oropharyngeal cancer. Cancer Prev Res (Phila).

[15] Lee SH, Lee CR, Rigas NK, Kim RH, Kang MK, Park NH, Shin KH (2015). Human papillomavirus 16 (HPV16) enhances tumor growth and cancer stemness of HPV-negative oral/oropharyngeal squamous cell carcinoma cells via miR-181 regulation. Papillomavirus Res.

[16] van Houten VM, Snijders PJ, van den Brekel MW, Kummer JA, Meijer CJ, van Leeuwen B, Denkers F, Smeele LE, Snow GB, Brakenhoff RH (2001). Biological evidence that human papillomaviruses are etiologically involved in a subgroup of head and neck squamous cell carcinomas. Int J Cancer.

[17] Fan KH, Wang HM, Kang CJ, Lee LY, Huang SF, Lin CY, Chen EY, Chen IH, Liao CT, Chang JT (2010). Treatment results of postoperative radiotherapy on squamous cell carcinoma of the oral cavity: coexistence of multiple minor risk factors results in higher recurrence rates. Int J Radiat Oncol Biol Phys.

[18] Liao CT, Wallace CG, Lee LY, Hsueh C, Lin CY, Fan KH, Wang HM, Ng SH, Lin CH, Tsao CK, Chen IH, Huang SF, Kang CJ (2014). Clinical evidence of field cancerization in patients with oral cavity cancer in a betel quid chewing area. Oral Oncol.

[19] McMahon J, O'Brien CJ, Pathak I, Hamill R, McNeil E, Hammersley N, Gardiner S, Junor E (2003). Influence of condition of surgical margins on local recurrence and disease-specific survival in oral and oropharyngeal cancer. Br J Oral Maxillofac Surg.

[20] Huang TY, Hsu LP, Wen YH, Huang TT, Chou YF, Lee CF, Yang MC, Chang YK, Chen PR (2010). Predictors of locoregional recurrence in early stage oral cavity cancer with free surgical margins. Oral Oncol.

[21] Fan KH, Wang HM, Kang CJ, Lee LY, Huang SF, Lin CY, Chen EY, Chen IH, Liao CT, Chang JT (2010). Treatment results of postoperative radiotherapy on squamous cell carcinoma of the oral cavity: coexistence of multiple minor risk factors results in higher recurrence rates. Int J Radiat Oncol Biol Phys.

[22] Montero PH, Yu C, Palmer FL, Patel PD, Ganly I, Shah JP, Shaha AR, Boyle JO, Kraus DH, Singh B, Wong RJ, Morris LG, Kattan MW (2014). Nomograms for preoperative prediction of prognosis in patients with oral cavity squamous cell carcinoma. Cancer.

[23] Wang HM, Liao CT, Yen TC, Chen SJ, Lee LY, Hsieh CH, Lin CY, Ng SH (2016). Clues toward precision medicine in oral squamous cell carcinoma: utility of next-generation sequencing for the prognostic stratification of high-risk patients harboring neck lymph node extracapsular extension. Oncotarget.

[24] Johnson JT, Myers EN, Bedetti CD, Barnes EL, Schramm VL, Thearle PB (1985). Cervical lymph node metastases. Incidence and implications of extracapsular carcinoma. Arch Otolaryngol.

[25] Kreimer AR, Johansson M, Waterboer T, Kaaks R, Chang-Claude J, Drogen D, Tjønneland A, Overvad K, Quirós JR, González CA, Sánchez MJ, Larrañaga N, Navarro C (2013). Evaluation of human papillomavirus antibodies and risk of subsequent head and neck cancer. J Clin Oncol.

[26] de Visscher AV, Manni JJ (1994). Routine long-term follow-up in patients treated with curative intent for squamous cell carcinoma of the larynx, pharynx, and oral cavity. Does it make sense?. Arch Otolaryngol Head Neck Surg.

[27] Halimi C, Barry B, De Raucourt D, Choussy O, Dessard-Diana B, Hans S, Lafarge D (2015). SFORL work-group. Guidelines of the French Society of Otorhinolaryngology (SFORL), short version. Diagnosis of local recurrence and metachronous locations in head and neck oncology. Eur Ann Otorhinolaryngol Head Neck Dis.

[28] Liao CT, Huang SF, Chen IH, Chang JT, Wang HM, Ng SH, Hsueh C, Lee LY, Lin CH, Cheng AJ, Yen TC (2008). When does skin excision allow the achievement of an adequate local control rate in patients with squamous cell carcinoma involving the buccal mucosa?. Ann Surg Oncol.

[29] Sinha P, Mehrad M, Chernock RD, Lewis JS, El-Mofty SK, Wu N, Nussenbaum B, Haughey BH (2015). Histologic and systemic prognosticators for local control and survival in margin-negative transoral laser microsurgery treated oral cavity squamous cell carcinoma. Head Neck.

[30] Park NH, Min BM, Li SL, Huang MZ, Cherick HM, Doniger J (1991). Immortalization of normal human oral keratinocytes with type 16 human papillomavirus. Carcinogenesis.

[31] Woo SB, Cashman EC, Lerman MA (2013). Human papillomavirus-associated oral intraepithelial neoplasia. Mod Pathol.

[32] Tachezy R, Klozar J, Rubenstein L, Smith E, Saláková M, Smahelová J, Ludvíková V, Rotnáglová E, Kodet R, Hamsíková E (2009). Demographic and risk factors in patients with head and neck tumors. J Med Virol.

[33] Ang KK, Harris J, Wheeler R, Weber R, Rosenthal DI, Nguyen-Tân PF, Westra WH, Chung CH, Jordan RC, Lu C, Kim H, Axelrod R, Silverman CC (2010). Human papillomavirus and survival of patients with oropharyngeal cancer. N Engl J Med.

[34] Anderson KS, Wong J, D'Souza G, Riemer AB, Lorch J, Haddad R, Pai SI, Longtine J, McClean M, LaBaer J, Kelsey KT, Posner M (2011). Serum antibodies to the HPV16 proteome as biomarkers for head and neck cancer. Br J Cancer.

[35] Anantharaman D, Gheit T, Waterboer T, Abedi-Ardekani B, Carreira C, McKay-Chopin S, Gaborieau V, Marron M, Lagiou P, Ahrens W, Holcátová I, Merletti F, Kjaerheim K (2013). Human papillomavirus infections and upper aero-digestive tract cancers: the ARCAGE study. J Natl Cancer Inst.

[36] O'Rorke MA, Ellison MV, Murray LJ, Moran M, James J, Anderson LA (2012). Human papillomavirus related head and neck cancer survival: a systematic review and meta-analysis. Oral Oncol.

[37] Chen AM, Zahra T, Daly ME, Farwell DG, Luu Q, Gandour-Edwards R, Vaughan AT (2013). Definitive radiation therapy without chemotherapy for human papillomavirus-positive head and neck cancer. Head Neck.

[38] O'Sullivan B, Huang SH, Siu LL, Waldron J, Zhao H, Perez-Ordonez B, Weinreb I, Kim J, Ringash J, Bayley A, Dawson LA, Hope A, Cho J (2013). Deintensification candidate subgroups in human papillomavirus-related oropharyngeal cancer according to minimal risk of distant metastasis. J Clin Oncol.

[39] Ramshankar V, Soundara VT, Shyamsundar V, Ramani P, Krishnamurthy A (2014). Risk stratification of early stage oral tongue cancers based on HPV status and p16 immunoexpression. Asian Pac J Cancer Prev.

[40] Saghravanian N, Zamanzadeh M, Meshkat Z, Afzal Aghaee M, Salek R (2016). Evaluation of the prevalence rate and the prognostic effect of human papilloma virus infection in a group of patients with oral cavity squamous cell carcinoma. Iran J Cancer Prev.

[41] Dediol E, Sabol I, Virag M, Grce M, Muller D, Manojlović S (2016). HPV prevalence and p16INKa overexpression in non-smoking non-drinking oral cavity cancer patients. Oral Dis.

[42] Lee LA, Huang CG, Tsao KC, Liao CT, Kang CJ, Chang KP, Huang SF, Chen IH, Fang TJ, Li HY, Yang SL, Lee LY, Hsueh C Increasing rates of low-risk human papillomavirus infections in patients with oral cavity squamous cell carcinoma: association with clinical outcomes. J Clin Virol.

[43] Haas I, Hauser U, Ganzer U (2001). The dilemma of follow-up in head and neck cancer patients. Eur Arch Otorhinolaryngol.

[44] Dahlstrom KR, Bell D, Hanby D, Li G, Wang LE, Wei Q, Williams MD, Sturgis EM (2015). Socioeconomic characteristics of patients with oropharyngeal carcinoma according to tumor HPV status, patient smoking status, and sexual behavior. Oral Oncol.

[45] Begum S, Cao D, Gillison M, Zahurak M, Westra WH (2005). Tissue distribution of human papillomavirus 16 DNA integration in patients with tonsillar carcinoma. Clin Cancer Res.

[46] Rietbergen MM, Braakhuis BJ, Moukhtari N, Bloemena E, Brink A, Sie D, Ylstra B, Baatenburg de Jong RJ, Snijders PJ, Brakenhoff RH, Leemans CR (2014). No evidence for active human papillomavirus (HPV) in fields surrounding HPV-positive oropharyngeal tumors. J Oral Pathol Med.

[47] Partlová S, Bouček J, Kloudová K, Lukešová E, Zábrodský M, Grega M, Fučíková J, Truxová I, Tachezy R, Špíšek R, Fialová A (2015). Distinct patterns of intratumoral immune cell infiltrates in patients with HPV-associated compared to non-virally induced head and neck squamous cell carcinoma. Oncoimmunology.

[48] Partridge M, Li SR, Pateromichelakis S, Francis R, Phillips E, Huang XH, Tesfa-Selase F, Langdon JD (2000). Detection of minimal residual cancer to investigate why oral tumors recur despite seemingly adequate treatment. Clin Cancer Res.

[49] Schache AG, Liloglou T, Risk JM, Filia A, Jones TM, Sheard J, Woolgar JA, Helliwell TR, Triantafyllou A, Robinson M, Sloan P, Harvey-Woodworth C, Sisson D (2011). Evaluation of human papilloma virus diagnostic testing in oropharyngeal squamous cell carcinoma: sensitivity, specificity, and prognostic discrimination. Clin Cancer Res.

[50] zur Hausen H (2002). Papillomaviruses and cancer: from basic studies to clinical application. Nature Rev Cancer.

[51] Wu DW, Chuang CY, Lin WL, Sung WW, Cheng YW, Lee H (2014). Paxillin promotes tumor progression and predicts survival and relapse in oral cavity squamous cell carcinoma by microRNA-218 targeting. Carcinogenesis.

[52] Sartor MA, Dolinoy DC, Jones TR, Colacino JA, Prince ME, Carey TE, Rozek LS (2011). Genome-wide methylation and expression differences in HPV(+) and HPV(−) squamous cell carcinoma cell lines are consistent with divergent mechanisms of carcinogenesis. Epigenetics.

[53] Tan HK, Saulnier P, Auperin A, Lacroix L, Casiraghi O, Janot F, Fouret P, Temam S (2008). Quantitative methylation analyses of resection margins predict local recurrences and disease-specific deaths in patients with head and neck squamous cell carcinomas. Br J Cancer.

[54] Yang B, Miao S, Zhang LN, Sun HB, Xu ZN, Han CS (2015). Correlation of CCNA1 promoter methylation with malignant tumors: a meta-analysis introduction. Biomed Res Int.

[55] Chawla JP, Iyer N, Soodan KS, Sharma A, Khurana SK, Priyadarshni P (2015). Role of miRNA in cancer diagnosis, prognosis, therapy and regulation of its expression by Epstein-Barr virus and human papillomaviruses: With special reference to oral cancer. Oral Oncol.

[56] Jithesh PV, Risk JM, Schache AG, Dhanda J, Lane B, Liloglou T, Shaw RJ (2013). The epigenetic landscape of oral squamous cell carcinoma. Br J Cancer.

[57] Van Doornum GJ, Korse CM, Buning-Kager JC, Bonfrer JM, Horenblas S, Taal BG, Dillner J (2003). Reactivity to human papillomavirus type 16 L1 virus-like particles in sera from patients with genital cancer and patients with carcinomas at five different extragenital sites. Br J Cancer.

[58] Smith EM, Ritchie JM, Pawlita M, Rubenstein LM, Haugen TH, Turek LP, Hamsikova E (2007). Human papillomavirus seropositivity and risks of head and neck cancer. Int J Cancer.

[59] Smeets SJ, Hesselink AT, Speel EJ, Haesevoets A, Snijders PJ, Pawlita M, Meijer CJ, Braakhuis BJ, Leemans CR, Brakenhoff RH (2007). A novel algorithm for reliable detection of human papillomavirus in paraffin embedded head and neck cancer specimen. Int J Cancer.

[60] Koslabova E, Hamsikova E, Salakova M, Klozar J, Foltynova E, Salkova E, Rotnaglova E, Ludvikova V, Tachezy R (2013). Markers of HPV infection and survival in patients with head and neck tumors. Int J Cancer.

[61] Dahlstrom KR, Anderson KS, Cheng JN, Chowell D, Li G, Posner M, Sturgis EM (2015). HPV serum antibodies as predictors of survival and disease progression in patients with HPV-positive squamous cell carcinoma of the oropharynx. Clin Cancer Res.

[62] Sarkar AK, Tortolero-Luna G, Follen M, Sastry KJ (2005). Inverse correlation of cellular immune responses specific to synthetic peptides from the E6 and E7 oncoproteins of HPV-16 with recurrence of cervical intraepithelial neoplasia in a cross-sectional study. Gynecol Oncol.

[63] Leemans CR, Braakhuis BJ, Brakenhoff RH (2011). The molecular biology of head and neck cancer. Nat Rev Cancer.

[64] Martinez-Zapien D, Ruiz FX, Poirson J, Mitschler A, Ramirez J, Forster A, Cousido-Siah A, Masson M, Vande Pol S, Podjarny A, Travé G, Zanier K (2016). Structure of the E6/E6AP/p53 complex required for HPV-mediated degradation of p53. Nature.

[65] Xie X, Piao L, Bullock BN, Smith A, Su T, Zhang M, Teknos TN, Arora PS, Pan Q (2014). Targeting HPV16 E6-p300 interaction reactivates p53 and inhibits the tumorigenicity of HPV-positive head and neck squamous cell carcinoma. Oncogene.

[66] Togtema M, Pichardo S, Jackson R, Lambert PF, Curiel L, Zehbe I (2012). Sonoporation delivery of monoclonal antibodies against human papillomavirus 16 E6 restores p53 expression in transformed cervical keratinocytes. PLoS One.

[67] Fakhry C, Qualliotine JR, Zhang Z, Agrawal N, Gaykalova DA, Bishop JA, Subramaniam RM, Koch WM, Chung CH, Eisele DW, Califano J, Viscidi RP (2016). Serum antibodies to HPV16 early proteins warrant investigation as potential biomarkers for risk stratification and recurrence of HPV-associated oropharyngeal cancer. Cancer Prev Res (Phila).

[68] Smeets SJ, van der Plas M, Schaaij-Visser TB, van Veen EA, van Meerloo J, Braakhuis BJ, Steenbergen RD, Brakenhoff RH (2011). Immortalization of oral keratinocytes by functional inactivation of the p53 and pRb pathways. Int J Cancer.

[69] Sivasithamparam J, Visk CA, Cohen EE, King AC (2013). Modifiable risk behaviors in patients with head and neck cancer. Cancer.

[70] Coppock JD, Wieking BG, Molinolo AA, Gutkind JS, Miskimins WK, Lee JH (2013). Improved clearance during treatment of HPV-positive head and neck cancer through mTOR inhibition. Neoplasia.

[71] Liao CT, Wang HM, Ng SH, Yen TC, Lee LY, Hsueh C, Wei FC, Chen IH, Kang CJ, Huang SF, Chang JT (2006). Good tumor control and survivals of squamous cell carcinoma of buccal mucosa treated with radical surgery with or without neck dissection in Taiwan. Oral Oncol.

[72] Neck Ebrahimi A, Gil Z, Amit M, Yen TC, Liao CT, Chaturvedi P, Agarwal JP, Kowalski LP, Kreppel M, Cernea CR, Brandao J, Bachar G, International Consortium for Outcome Research (ICOR) in Head Cancer (2014). Primary tumor staging for oral cancer and a proposed modification incorporating depth of invasion: an international multicenter retrospective study. JAMA Otolaryngol Head Neck Surg.

[73] Greene FL, Page DL, Fleming ID, Fritz A, Balch CM, Haller DG, Morrow M (2002). AJCC Cancer Staging Manual.

[74] Chao FY, Chao A, Huang CC, Hsueh S, Yang JE, Huang HJ, Wang LC, Lin CT, Chou HH, Lai CH (2010). Defining detection threshold and improving analytical proficiency of HPV testing in clinical specimens. Gynecol Oncol.

[75] von Ahlfen S, Missel A, Bendrat K, Schlumpberger M (2007). Determinants of RNA quality from FFPE samples. PLoS One.

[76] Chang JT, Wong FH, Liao CT, Chen IH, Wang HM, Cheng AJ (2004). Enzyme immunoassay for serum autoantibody to survivin and its findings in head-and-neck cancer patients. Clin Chem.

[77] Chang KP, Kao HK, Liang Y, Cheng MH, Chang YL, Liu SC, Lin YC, Ko TY, Lee YS, Tsai CL, Wang TH, Hao SP, Tsai CN (2010). Overexpression of activin A in oral squamous cell carcinoma: association with poor prognosis and tumor progression. Ann Surg Oncol.

[78] Chen HH, Chen IH, Liao CT, Wei FC, Lee LY, Huang SF (2011). Preoperative circulating C-reactive protein levels predict pathological aggressiveness in oral squamous cell carcinoma: a retrospective clinical study. Clin Otolaryngol.

[79] Munkhdelger J, Choi Y, Lee D, Kim S, Kim G, Park S, Choi E, Jin H, Jeon BY, Lee H, Park KH (2014). Comparison of the performance of the NucliSENS EasyQ HPV E6/E7 mRNA assay and HPV DNA chip for testing squamous cell lesions of the uterine cervix. Diagn Microbiol Infect Dis.

[80] Ferguson M, Wilkinson DE, Heath A, Matejtschuk P (2011). The first international standard for antibodies to HPV 16. Vaccine.

[81] Efron B, Tibshirani RJ (1994). An introduction to the bootstrap. Boca.

[82] Heagerty PJ, Lumley T, Pepe MS (2000). Time-dependent ROC curves for censored survival data and a diagnostic marker. Biometrics.

[83] Steyerberg EW, Eijkemans MJ, Harrell FE, Habbema JD (2001). Prognostic modeling with logistic regression analysis: in search of a sensible strategy in small data sets. Med Decis Making.

[84] Babyak MA (2004). What you see may not be what you get: a brief, nontechnical introduction to overfitting in regression-type models. Psychosom Med.

[85] Faul F, Erdfelder E, Buchner A, Lang AG (2009). Statistical power analyses using G*Power 3.1: tests for correlation and regression analyses. Behav Res Methods.

